# MAMMA MIA! Norwegian Midwives’ Practices and Views About Gestational Weight Gain, Physical Activity, and Nutrition

**DOI:** 10.3389/fpsyg.2020.01463

**Published:** 2020-07-24

**Authors:** Lene A. H. Haakstad, Julie M. F. Mjønerud, Emilie Mass Dalhaug

**Affiliations:** Department of Sports Medicine, Norwegian School of Sport Sciences, Oslo, Norway

**Keywords:** gestational weight gain, lifestyle counseling, midwives, nutrition, physical activity, pregnancy, prenatal care

## Abstract

**Objectives:**

Most studies regarding prevalence of prenatal lifestyle counseling are based on patient report of provider advice. The aim of the present study was to describe midwives’ practice and views in promoting three distinct, but importantly related lifestyle factors: gestational weight gain (GWG), regular physical activity (PA), and nutrition.

**Design:**

A cross-sectional study.

**Setting:**

Healthcare clinics in Oslo and Akershus County, Norway.

**Participants:**

Clinics that expressed interest to participate provided an email list of the midwives. Of 107 midwives invited to participate, 65 completed the 15-min electronic survey (SurveyXact), giving a response rate of 60.7%.

**Outcome Measures:**

We developed a new questionnaire based on questions and results from similar studies, as no validated questionnaires existed when we initiated this project in 2014. The final electronic questionnaire included a mix of close-ended questions, semi–close-ended questions, and 11-point Likert scales and covered demographics, personal health behaviors, counseling practice, views, and self-perceived role in lifestyle counseling.

**Results:**

Mean workload in prenatal care was 78%, and mean years practicing was 8.9 (±7.5). Across all three health topics, most (74–95%) reported to give advice on the first meeting, with a mean frequency of 2.2 (±1.4), 2.7 (±1.8), and 2.7 (±2.0) for GWG, PA, and nutrition counseling, respectively. Approximately 40% did not report advice on GWG or give advice discordant with the Institute of Medicine (IOM) recommendations (2009) for at least one prepregnancy body mass index (BMI) category. GWG was rated as more unpleasant to talk about than PA (3.0 ± 2.8 vs. 1.1 ± 2.5, *p* < 0.001) and nutrition (3.0 ± 2.8 vs. 1.2 ± 2.5, *p* = 0.002). Also, regarding the importance of giving lifestyle advice, PA (9.6 ± 0.9 vs. 8.3 ± 2.2, *p* < 0.001) and nutrition (9.9 ± 0.4 vs. 8.3 ± 2.2, *p* < 0.001) were rated as more important than advice about GWG. Postpartum, nearly 40% gave advice about PA, whereas only two (3.1%) reported to discuss weight/weight retention (*p* < 0.001).

**Conclusion:**

While most midwives gave advice on GWG, PA, and nutrition at the first meeting and rated lifestyle counseling as an important topic, the advice on GWG was often discordant with IOM recommendations, and the topic was viewed as more unpleasant to talk about than PA and nutrition.

## Introduction

It is suggested that important life transitions, including medical diagnosis or other naturally occurring events, such as pregnancy and entering parenthood, may motivate individuals to adopt, change, and maintain health-enhancing behaviors (McBride et al., 2003). Having a baby is a major life event, and for the majority of women, pregnancy is a period when they have sustained contact with a healthcare provider (Shieh et al., 2010). In Norway, prenatal care is free of charge and generally provided through 8–12 alternating visits with midwives and general practitioners, including an ultrasound scan (Backe, 2001; The Norwegian Directorate of Health, 2019)^1^. This regular counseling throughout gestation, and that women may be motivated to make healthy lifestyle changes, has suggested pregnancy to be a “window of opportunity” (ACOG, 2015). A recently published systematic review and meta-analysis of individual participant data from 36 trials (*n* = 12,526) showed that women randomized to lifestyle intervention and exercise had slightly lower average gestational weight gain (GWG) (−0.70 kg) compared to women in the control group (International Weight Management in Pregnancy (i-Wip) Collaborative Group, 2017). This is in accordance with others reviews (da Silva et al., 2017; Goldstein et al., 2017), favoring the intervention group compared with the controls (−0.9 to −1.1 kg). Similar evidence was found in overweight or obese populations (Choi et al., 2013; Farpour-Lambert et al., 2018). In addition, in high-income nations, 80–90% of women have at least one child during their reproductive years (Rostad et al., 2006; Martinez et al., 2012). Hence, healthcare providers may have a widespread opportunity to capitalize on this increased motivation by promoting positive behavior change.

The Institute of Medicine (IOM) (Institute of Medicine [IOM], 2009) has summarized substantial literature on GWG, including several cohort studies assessing GWG on pregnancy outcomes for women who are overweight or obese. The results show that, compared with higher and lower weight gain, GWG within the recommended range is associated with a reduced risk of maternal and infant complications (Institute of Medicine [IOM], 2009). Still, there are no formal, evidence-based guidelines from Norway or other European countries, and it is important to highlight that the IOM guidelines are largely based on observational data (Siega-Riz et al., 2009).

Regular physical activity (PA) and healthy eating may lead to adequate GWG and prevent postpartum weight retention and women’s future risk of overweight and obesity, also with reference to women who already have a high body mass index (BMI) and their weight management (ACOG, 2013). In addition, a healthy lifestyle may reduce the risks of adverse pregnancy and birth outcomes, including gestational diabetes mellitus, gestational hypertension and preeclampsia, depressive symptoms, preterm birth, macrosomia, small for gestational age infants, and cesarean deliveries (ACOG, 2015; Mottola et al., 2018; Dipietro et al., 2019). Hence, it is important that women receive consistent and updated counseling on these topics during pregnancy (ACOG, 2015).

The American College of Obstetricians and Gynecologists issued a committee opinion report in 2013 (ACOG, 2013), emphasizing that healthcare providers should consider prepregnancy BMI at the first prenatal visit, with the aim to advice on appropriate GWG, PA/exercise, and nutrition throughout gestation. Correspondingly, The Norwegian Directorate of Health (2019) recommends that all pregnant women should receive lifestyle counseling on these topics. Still, studies indicate that provider–patient communication regarding maternal health behavior is inadequate, largely limited by lack of knowledge or skills to undertake this type of counseling, in addition to be of low priority (Stengel et al., 2012; Willcox et al., 2012; Wilkinson et al., 2013; Morris et al., 2017; McGee et al., 2018). Current data show a wide range in numbers receiving GWG advice (20–60%) and that women with obesity were more likely than normal-weight women to receive such guidance (Whitaker et al., 2016; Morris et al., 2017; Vinturache et al., 2017; Wilkinson et al., 2017). The factors influencing GWG counseling are many-sided, and it is important to also acknowledge the psychological aspects (such as affect, cognition, and personality) that could explain the dialog between the healthcare provider and the pregnant women (Kapadia et al., 2015). Hitherto, no studies have explored these associations.

McGee et al. (2018) reported that fewer than 25% of obstetricians regularly discussed exercise during prenatal care, whereas others have found somewhat higher numbers (30–60%) with respect to combined PA/nutrition counseling (Whitaker et al., 2016; Santo et al., 2017; Vinturache et al., 2017). Up to date, most studies regarding the prevalence of prenatal lifestyle counseling are based on the women’s report of provider advice and the vast majority are done in the United States or Canada (Whitaker et al., 2016; Morris et al., 2017; Santo et al., 2017; Vinturache et al., 2017; Emery et al., 2018; McGee et al., 2018), except from a body of work on the management of GWG from Australia (Wilkinson et al., 2013; de Jersey et al., 2018, 2019). In many European countries, like Norway, midwives provide approximately 50% of the antenatal care (Backe, 2001; The Norwegian Directorate of Health, 2019, see text footnote 1), whereas this is far less prevalent especially in the United States (Vedam et al., 2018). Women report receiving different advice from obstetricians, general practitioners, and midwives (Ferrari et al., 2013), and the time of the visits varies, typically 10, 15, and 30 min, respectively (McDonald et al., 2012). Hence, the duration of these appointments may influence the amount of time available for counseling on lifestyle topics. Considering the above and that little is known about midwife-based prenatal care in Norway, including to what extent pregnant women are advised on GWG, PA, and nutrition, the primary aim of the present cross-sectional study was to describe midwives’ practice and views about these three health topics.

## Materials and Methods

### Study Design and Participants

This was a cross-sectional study design, using an electronic questionnaire (a paper version was also available) to investigate midwives’ practice and views about GWG, PA/exercise, and nutrition, conducted in Oslo and Akershus County, Norway.

Oslo and Akershus are highly populated counties in Norway, comprising both urban and rural settings, with 45 healthcare clinics (Oslo: 17 and Akershus: 28) and 130 midwives working with prenatal care. The project manager called all healthcare clinics in Oslo and Akershus and talked to the head of the clinics. Midwives were considered eligible if they were seeing prenatal woman at the time of study enrollment. The clinics that expressed interest to participate received additional information about the project and responded back with an email list of the midwives working at the respective clinic. Because of restrictions of privacy, not all health clinics (*n* = 12) agreed to distribute email contact information. Of 107 invited midwives, 65 completed the questionnaire, giving a response rate of 60.7%. Unfortunately, we do not have any data or explanations about why certain health clinics did not want to participate in the study and are aware that this may have given intrinsic bias.

### Ethical Approval

The study was reviewed by the Regional Committee for Medical and Health Research Ethics (REK 2015/1941 A), who concluded that, according to the Act on medical and health research (the Health Research Act 2008), the study did not require full review by REK. The study was approved by the Norwegian Social Science Data Services (NSD 560627). Following the Helsinki Declaration, the first survey page included in-depth information about the study’s purpose, procedures, and outcomes. It was emphasized that participation was voluntary and that they could withdraw from the project at any time with no explanation required. Hence, to complete the online survey, the midwives had to confirm that they had received adequate information and wanted to participate by checking the associated box. No financial support was given.

### Data Collection

The electronic survey (SurveyXact) was sent by e-mail directly from the project manager to the midwives and then automatically forwarded back to us after completion. After approximately 2 weeks, one reminder was sent to those who had not responded. The respondent could not change the answers after completion. One clinic wanted to use a paper version of the survey, and the head of the clinic handed out questionnaires to the midwives (*n* = 10). Two weeks later, the project manager picked up four completed surveys from these clinics.

### Outcome Measures

To our knowledge, no validated questionnaires on practices of the healthcare provider existed when we initiated this project in 2014. Hence, we developed a new questionnaire based on questions and results from similar studies (Entin and Munhall, 2006; Bauer et al., 2010; Chang et al., 2013). Both the electronic questionnaire and paper version included 72 questions, required approximately 15 min to complete, and were a mix of close-ended questions, semi–close-ended questions, and 11-point Likert scales, divided into seven subcategories. No validation was performed for this questionnaire; however, all question-and-answer options were piloted for comprehensibility among six midwives, as well as among the research group, and were revised accordingly. We will also highlight that future studies addressing midwives’ practices and views about GWG, PA, and nutrition should include more information about personal lifestyle choices, including height and body weight for calculation of participants’ BMI, as well as parity, valuable in assessing if this influenced lifestyle advice.

Following is a presentation of the four subcategories, 26 questions, and three statements used to answer the present research questions.

#### Participants’ Demographics

Participants’ demographics addressed age, gender, number of years practicing antenatal care, and percentage of workload consisting of antenatal care.

#### Personal Health Behaviors

Providers’ personal behaviors regarding PA, diet, and smoking were assessed with the following questions (Haskell et al., 2007; Nordic Nutrition Recommendations, 2014):

• All adults are recommended to perform moderate-intensity PA (activities that make you breathe somewhat harder than normal, such as brisk walking, housework, etc.) for a minimum of 30 min 5 days a week, equal to 2.5 h. With this in mind, would you characterize yourself as physically active?
  Response options: “yes,” “no,” or “I don’t know.”
• The Norwegian Directorate of Health^2^ recommends a balanced and varied diet, comprising whole-grain products, vegetables, fruits and berries, lean dairy products, fish, legumes, and nuts, while also limiting the amount of processed meats, red meat, and foods high in saturated fat, sugar, and salt. With this in mind, how would you characterize your diet in the current week?
  The participants rated their diet on a scale from 0 to 10, where 0 represented *“*very bad,” and 10 represented “excellent.” According to these levels, the responses were also divided into two categories: healthy eating habits (excellent and good diet: ≥8) and unhealthy eating habits (average, bad and very bad diet: <8).
• Do you smoke daily?
  Response options: “yes” or “no”.

#### Counseling Practice

Whether or not the midwives gave advice to their pregnant women on GWG, PA, and nutrition was assessed using a simple yes or no question for each topic. Providers answering yes were asked to elaborate on what they based their advice on. The categorical responses developed for this study were as follows: “Recommendations from the Norwegian Directorate of Health (see text footnote 2),” “Research articles/scientific evidence,” “Supplementary education/conferences,” and “Own experiences.” Participants had the opportunity to choose more than one response category.

Frequency (“How often do you give advice/inform about GWG, PA, and nutrition?”) and when such advices were given throughout gestation (for each of the three health topics) were also examined. For the latter, the midwives could choose more than one categorical option: “first visit,” “first trimester,” “second trimester,” “third trimester,” “postpartum,” and/or “at all occasions.”

To evaluate if the advice regarding GWG was coherent with the IOM recommendations (2009), we asked the following questions: “Based on a women’s prepregnancy BMI category, how much (total GWG in kg) would you recommend her to gain during pregnancy?: (1) underweight, (2) normal weight, (3) overweight, and (4) obese.” Responses were given as one value.

#### Views

If the midwives answered no to counseling on GWG, PA, and nutrition, the reasons why were investigated. The categorical responses were as follows: “I do not have the time to address lifestyle behaviors,” “Physical activity/nutrition/weight gain is of low priority in the context of a typical prenatal visit,” “I do not have sufficient knowledge/skills regarding physical activity/nutrition/weight gain,” and “The women are not interested in talking about these topics.”

Further, to explore self-perceived role in promoting lifestyle factors, the participants were asked to rate three different statements for each health topic:

• “To give pregnant women advice on weight gain/physical activity/nutrition/is a very important part of prenatal care.”
• “It is uncomfortable/difficult to talk to pregnant women about physical activity/nutrition/weight gain.”
• “For pregnant women and her baby, appropriate weight gain/physical activity/a healthy diet/is of great importance.”

Responses indicated level of agreement with each statement on a scale from 0 (strongly disagree) to 10 (strongly agree). Response options and statements were based on results from similar studies (Chang et al., 2013; Morris et al., 2017).

### Statistical Analysis

All statistical analyses were conducted with SPSS software version 24 for Windows. Data were presented as numbers with percentages or means with standard deviation and *p* values. χ^2^ analysis was used to compare categorical data and two-sided independent-sample *t* test for continuous data. Differences in mean scores for self-perceived role in lifestyle counseling were compared using one-way analysis of variance.

All descriptive data were explored for normality and determined by skewness, histograms, and significance level (Kolmogorov–Smirnov test for normality) in SPSS. The histogram was emphasized if the three variables showed both normality and skewness. Even though some of the data were not normally distributed, we chose after discussion with professor in biostatistics (Morten Vang Fagerland, Head of Section for Biostatistics and Epidemiology, Oslo University Hospital, Norway), to compare differences using parametric tests because of the sample size (*n* ≥ 30) (Pallant, 2013).

As most respondents reported a healthy lifestyle with respect to PA (88%) and healthy eating habits (68%), the numbers would have been too small to investigate the associations between poor personal health behavior and whether they provided counseling on GWG, PA, and nutrition. In addition, we did not ask about providers’ body weight and are therefore not able to assess this as a as predictor of GWG advice.

## Results

### Participant Characteristics

Of the 65 midwives participating in the study, 64 were women, and mean age was 47.5 years (±8.9). Mean workload in prenatal care was 78%, and mean years practicing was 8.9 (±7.5). Eighty-eight percent reported to be regularly active according to guidelines; 68% had high adherence to nutritional recommendations, and none used tobacco daily (Table 1).

**TABLE 1:** General characteristics and personal health behaviors of the participants (*n* = 65).

**Variable**	
Age in years [Mean (SD*)]	47.5 (8.9)
Gender [n (%)]	
Women	64 (98.5)
Years practicing [Mean (SD)]	8.9 (7.5)
<10 years [n (%)]	37 (57.8)
≥10 years [n (%)]	27 (42.2)
Proportion of prenatal care [n (%)]	
<50%	6 (9.4)
≥50%	58 (90.6)
Location of health clinic [n (%)]	
Oslo	36 (55.4)
Akershus	29 (44.6)
Current daily smoker [n (%)]	0
Units of alcohol weekly [n (%)]	
None	11 (17.2)
1–5 (light drinking)	49 (76.6)
6–13 (moderate drinking)	3 (4.7)
≥14 (heavy drinking)	1 (1.6)
Healthy eating habits [n (%)]	44 (67.7)
Regular PA active** [n (%)]	57 (87.7)

*SD; Standard Deviation, **Moderate-intensity physical activity (PA) ≥ 2.5 h weekly.

### Practice and Views About Lifestyle Variables and Health Behaviors

All the midwives reported giving advice on PA at least once throughout gestation, whereas five (7.7%) and four (6.2%) reported no counseling on nutrition and GWG, respectively (Table 2). One answered that GWG advice is of low priority in the context of a typical prenatal visit; otherwise, no other reasons for not providing advice were reported.

**TABLE 2:** Practice and views about lifestyle variables and health behaviors (*n* = 65).

**Variable**	**GWG**	**PA**	**Nutrition**
Advice ≥ once throughout gestation [n (%)]	61 (93.8)	65 (100)	60 (92.3)
Frequency of counseling [Mean (SD)]	2.2 (1.4)	2.7 (1.8)	2.7 (2.0)
1–3 talks	35 (53.8)	48 (80.0)	42 (64.6)
4–6 talks	9 (13.8)	9 (15.0)	6 (9.2)
≥7 talks	0 (0)	3 (5.0)	5 (7.7)
Counseling was given at. *[n (%)]			
First visit	48 (73.8)	62 (95.4)	53 (81.5)
1st trimester	14 (21.5)	21 (32.3)	15 (23.1)
2nd trimester	18 (27.7)	27 (41.5)	17 (26.2)
3rd trimester	14 (21.5)	15 (23.1)	13 (20.0)
Postpartum	2 (3.1)	24 (36.9)	11 (16.9)
At all occasions	18 (27.7)	17 (26.2)	21 (32.3)
Basis/source for the advice* [n (%)]			
Health authorities	59 (90.8)	63 (96.9)	63 (96.9)
Research/scientific evidence	23 (35.4)	37 (56.9)	29 (44.6)
Supplementary education/conferences	7 (10.8)	6 (9.2)	13 (20.0)
Own experiences	2 (3.1)	21 (32.3)	8 (12.3)
Handing out information pamphlets	26 (40.0)	40 (61.5)	49 (75.4)
Self-perceived role in lifestyle counseling ** [Mean (SD)]			
*“. to give advice is an important part of prenatal care”*	8.3 (2.2)	9.6 (0.9)	9.9 (0.4)
*“. it is uncomfortable to talk about”*	3.0 (2.8)	1.1 (2.5)	1.2 (2.5)
*“. GWG/PA/nutrition is important for a healthy pregnancy”*	7.1 (3.0)	9.9 (0.4)	9.8 (0.6)

*Selection of more than one response was allowed, and the participants were instructed to tick one or more alternatives that best matched their practice, **Responses indicated level of agreement with each statement on a scale from 0 (strongly disagree) to 10 (strongly agree).

Across all three health topics, most midwives reported to give advice on the first visit, with approximately one-quarter following up this counseling throughout gestation. Frequency of counseling was approximately twice for GWG, and three times for PA and nutrition (Table 2). Postpartum, nearly 40% gave advice about PA, whereas only two (3.1%) reported to talk about weight/weight retention (*p* < 0.001).

When viewing all three lifestyle factors as one, the vast majority of providers reported basing their advice on recommendations from the health authorities and a brochure published by the Norwegian Directorate of Health^3^ (Table 2). In addition, regarding PA counseling, 32.3% based their advice on personal sport/exercise experiences, whereas 3.1% and 12.2% used their own experiences to give advice on GWG and nutrition, respectively (*p* = 0.006). We do not have any data about numbers of midwives who had been pregnant and cannot further investigate if their own pregnancy experiences were important for the information provided.

The midwives rated dialog about GWG as more uncomfortable/difficult than discussions about PA (*p* < 0.001) and nutrition (*p* = 0.002). Also, regarding the importance of giving lifestyle advice during consultations, PA (*p* < 0.001) and nutrition (*p* < 0.001) were rated as more important subjects than giving advice about GWG (Table 2).

### Gestational Weight Gain

Nearly 40% of the midwives did not report or give advice on GWG discordant with the IOM recommendations (2009) for at least one prepregnancy BMI category. The proportion of providers giving advice consistent with the guidelines did not differ between the prepregnancy BMI categories, and mean values were within the recommended range for all groups (Table 3).

**TABLE 3:** Midwives recommendations of GWG, using pre-pregnancy BMI groupings recommended by the Institute of Medicine (IOM) (*n* = 65).

**Variable**	**Below**	**Within**	**Above**	**No response**
Underweight women (pre-pregnancy BMI < 18.5)	11 (16.9)	35 (53.8)	2 (3.1)	17 (26.2)
Recommended GWG in kg [Mean (SD)]	14.8 (2.3)			
Normal weight women (pre-pregnancy BMI 18.5–24.9)	9 (13.8)	39 (60.0)	0	17 (26.2)
Recommended GWG in kg [Mean (SD)]	12.7 (1.7)			
Overweight women (pre-pregnancy BMI 25.0–29.9)	5 (7.7)	41 (63.1)	1 (1.5)	18 (27.7)
Recommended GWG in kg [Mean (SD)]	8.8 (1.7)			
Obese women (pre-pregnancy BMI ≥ 30)	6 (9.2)	37 (56.9)	4 (6.2)	18 (27.7)
Recommended GWG in kg [Mean (SD)]	6.1 (2.5)			

*Results are given in numbers (%), unless otherwise is specified.*

## Discussion

Most midwives reported counseling pregnant women about GWG, PA, and nutrition at first prenatal visit. Still, repeated lifestyle counseling is not met as recommended by ACOG (2013) and the Norwegian health authorities (The Norwegian Directorate of Health, 2019). In the present study, few routinely provided women with advice about GWG, PA, and nutrition, and merely one-quarter gave advice about these topics more than once throughout gestation. GWG was viewed as more uncomfortable to talk about than PA and nutrition. Also, regarding the importance of giving lifestyle advice, PA and nutrition were rated as more important than advice about GWG, and 40% did not report or give advice on GWG discordant with the present recommendations for at least one prepregnancy BMI category.

The low rates of midwives answering the specific section about recommended total weight gain are of concern, because it is likely that non-respondents are those who do not give advice or are not familiar with the IOM guidelines. Postpartum, only two reported talking about weight/weight retention, compared with 36.9% and 16.9% having a dialog with the women about PA and nutrition, respectively. Hence, more midwives used this opportunity to talk about lifestyle choices, PA and healthy eating, which may lead to healthy body weight, rather than attention on weight management.

In Australia, 66% reported that they needed more training in counseling and that insufficient time was a main barrier to conversing with women (de Jersey et al., 2019). In addition, our results are in contrast to what women state that they want during prenatal consultations, as studies conclude that most women would prefer GWG advice early, with regular follow-ups and discussions during the course of pregnancy, as well as postpartum (de Jersey et al., 2013; Nikolopoulos et al., 2017). Studies have also shown that prenatal nutrition and related lifestyle counseling throughout gestation may lower GWG and neonatal macrosomia, especially in a high-risk population, such as women with overweight or obesity (Mitchell et al., 2017; Peccei et al., 2017). Hence, healthcare providers have the potential to influence and be a reliable source of evidence-based health information during an important period of a woman’s life (Weeks et al., 2018). de Jersey et al. (2018) found that a brief education session integrated into an existing mandatory training program has shown positive results in improving the knowledge and confidence of midwives in delivering advice and support for healthy GWG. Midwifery training could potentially also be a solution to this problem in Norway and better prepare midwives to discuss issues related to weight management during pregnancy and postpartum (Premji et al., 2019).

### Strengths and Weaknesses of the Study

One strength of the present study is the response rate and sample size, comprising a large proportion (50%) of all midwives working in Oslo and Akershus, two highly populated counties in Norway, comprising both urban and rural settings. Hence, our results have good generalizability, compared with most other research in this area, using qualitative methods with a small sample size (Willcox et al., 2012; Chang et al., 2013; Wennberg et al., 2014; Whitaker et al., 2016; Arrish et al., 2017). Also compared to other email surveys (Lutsiv et al., 2012; Ferraro et al., 2013; McGee et al., 2018), our sample size and response rate may be considered high. Furthermore, we adapted already validated questions and questions used in similar populations (Chang et al., 2013; Morris et al., 2017), and pilot-tested the survey before use.

This is the first study in Norway to describe midwives’ practice with respect to lifestyle counseling on three distinct, but importantly related topics: GWG, PA, and nutrition, and also one of the first to report on midwives’ knowledge of appropriate GWG, in conjunction with IOM guidelines (Institute of Medicine [IOM], 2009). Still, the results may only be generalizable to Norway.

While we were able to describe whether the midwives reported giving lifestyle advice, we could not evaluate the quality of the dialogue and information presented to the pregnant women. In addition, all data were self-reported, and the participants were aware of study aims; hence, we cannot rule out social desirability bias, or the “open book strategy,” meaning that the responders checked the IOM weight gain guidelines before giving responses. Nevertheless, if this was the case, the results of the present study provide a conservative calculation of midwives’ practice and views of GWG, PA, and nutrition.

### Discussion of Results

In high-income nations, the average number of prenatal consultations is 8–10, with only a slight difference between nulliparous and multiparous women (Backe, 2001; Stephansson et al., 2018). This frequent counseling throughout gestation and also that women may be more receptive and motivated to make changes for their own and their babies health have suggested pregnancy to be a particular “teachable moment” (ACOG, 2015). Still, and in line with the present results, qualitative research and surveys have shown that provider–patient communication regarding GWG, PA, and nutrition is infrequent and that, unless requested, few pregnant women receive regular guidance about these health topics (de Jersey et al., 2012, 2013; Stengel et al., 2012; Willcox et al., 2012; Whitaker et al., 2016; Morris et al., 2017; Wilkinson et al., 2017; Emery et al., 2018; McGee et al., 2018; Dalhaug and Haakstad, 2019). Hence, this evidence signals a lack of focus on lifestyle behavior in pregnancy and highlights the importance of improving healthcare provider’s knowledge, confidence, and skills in giving such guidance. On the other side, most of the studies regarding prevalence of prenatal lifestyle counseling are based on the pregnant women’s report of provider advice (Stengel et al., 2012; Whitaker et al., 2016; Emery et al., 2018; Dalhaug and Haakstad, 2019), and some have shown little congruence between patient and healthcare providers perceptions of counseling practice (Lutsiv et al., 2012; Ferraro et al., 2013). This discrepancy may be due to the healthcare providers giving socially desirable responses or the pregnant women not recalling having received advice from their healthcare provider.

Compared with women counseled by obstetricians or general practitioners, a higher proportion seeing midwives reported having discussed GWG, PA, and nutrition (35–39% vs. 64%) (McDonald et al., 2012; Premji et al., 2019). Also, midwives’ self-report in promoting lifestyle counseling shows more frequent lifestyle advice compared to other healthcare providers (Lutsiv et al., 2012; Ferraro et al., 2013; Morris et al., 2017). Hence, there seems to be some evidence that midwives as a group discuss health-enhancing behaviors more often than other prenatal providers do. There is, however, a need for educational input to enhance the quality of information and confidence in delivering such advice for all healthcare providers, including the midwives.

Our results are consistent with the results of McDonald et al. (2012), showing that approximately 60% of pregnant women had received advice about appropriate GWG as recommended by IOM from their midwife. Still, a significant proportion of pregnant women do not receive evidence-based advice about appropriate GWG, and this lack of information may drive several pregnant women to seek other information sources such as internet, blogs, online forums, books, parenting magazines, family, and friends, often being less reliable (McDonald et al., 2012; Dalhaug and Haakstad, 2019). In addition, health professionals who view lifestyle counseling as important are likely those who respond to a survey related to GWG, PA, and nutrition, hence overestimating the rate of giving such advice in the present study, and as such being a potential source of bias (Morris et al., 2017).

### Explanations and Implications

There are many competing interests during prenatal consultations. Providers are required to assess medical, familial, pregnancy, and psychological history, as well as provide information, antenatal tests, procedures, and bookings (Willcox et al., 2012). Stengel et al. (2012) found that pregnant women often only received lifestyle advice from their provider at the initial prenatal visit and that it sometimes was limited to written education. On the other side, information leaflet may be valuable and a source that can be referred to and used by the women in the future.

The midwives in our study gave, on average, advice on GWG, PA, and nutrition between two and three times, including the postpartum visit. Also, a large proportion handed out information pamphlets, especially for healthy eating during pregnancy. However, approximately only 23% followed up advice on GWG and nutrition in all trimesters. We do not know why the midwives in the present study did not prioritize lifestyle counseling on a regular basis, as only one participant gave response to the question addressing this: “Why do you not prioritize to advice on lifestyle variables: GWG, PA, and nutrition?” Hence, this is a flaw in the questionnaire, and if other researchers should address the same issue, the question must be rephrased to address potential barriers (e.g., “What do you perceive as the most important barriers with respect to give lifestyle counseling?”). Others have reported that lack of time and not perceiving it as important are key barriers to advising pregnant women on GWG, PA, and nutrition (Willcox et al., 2012; Whitaker et al., 2016).

Providers in our study rated PA and nutrition as more important for a healthy pregnancy than favorable weight gain. These results agree with the studies of Willcox et al. (2012) and Chang et al. (2013), both showing that although management of GWG was given a low priority, most providers recognized the importance of diet and exercise. Sensitivity of the topic and the providers feeling uncomfortable when talking to women about GWG, knowing that some women may feel offended or embarrassed, could also be a barrier to discussing weight-related issues. Consistent with previous research (Chang et al., 2013; Whitaker et al., 2016), the midwives in our study reported GWG to be slightly unpleasant to talk about. Another study also found that midwives have expressed concern for the trend that many pregnant women are worried about putting on too much weight, and in order not to make women more anxious, midwives choose to avoid the topic (Willcox et al., 2012). It can be discussed whether it is acceptable that midwives avoid dialog about GWG and regardless of whether the women are afraid of gaining too much or, on the other side, need to limit GWG. Either way, given the importance of appropriate GWG, this should be properly addressed. Hence, responses to our survey, as well as other research (Willcox et al., 2012), indicate missed opportunities in information exchange, as well as a need to improve midwives’ confidence and knowledge in giving GWG guidance.

### Future Research

Upcoming studies should evaluate more in-depth the quality of lifestyle counseling, including knowledge, beliefs, and attitudes of healthcare providers, as well as elucidate the effectiveness of different intervention approaches to increase the number of women who are accurately and effectively advised about GWG, PA, and nutrition during pregnancy. Research is also needed to evaluate midwife-based prenatal care compared with general practitioners.

### Conclusion

Few midwives routinely provided advice about GWG, PA, and nutrition, and merely one-quarter addressed these topics more than once throughout gestation. Hence, lifestyle counseling seems to be of low priority in the context of a typical prenatal visit. In addition, a high percentage did not provide data or give values on GWG discordant with the present IOM guidelines, as well as viewed GWG as more unpleasant to talk about than PA and nutrition. Given the importance of appropriate GWG, regular PA, and a healthy diet during pregnancy, as well as frequent and sustained contact between providers and pregnant women, the present results support the “window of opportunity” hypothesis. We further highlight that midwives need guidelines and education to play a more active role in lifestyle counseling. Studies are also warranted to increase our understanding of psychological factors associated with GWG guidance.

## Data Availability Statement

The datasets generated for this study are available on request to the corresponding author.

## Ethics Statement

The studies involving human participants were reviewed and approved by the Regional Committee for Medical and Health Research Ethics (REK 2015/1941 A), who concluded that, according to the Act on medical and health research (the Health Research Act 2008), the study did not require full review by REK. The study was approved by the Norwegian Social Science Data Services (NSD 560627). The patients/participants provided their written informed consent to participate in this study.

## Author Contributions

LH and ED conceived the idea for the study and developed the survey questionnaire. JM and ED were responsible for participant follow-up and data collection. LH supervised the project and outlined the manuscript. All authors read and corrected draft versions of the manuscript and approved the final version.

## Conflict of Interest

The authors declare that the research was conducted in the absence of any commercial or financial relationships that could be construed as a potential conflict of interest.

The handling editor declared a past co-authorship with one of the authors, LH.

## Footnotes

1 https://www.helsedirektoratet.no/retningslinjer/svangerskapsomsorgen/konsultasjoner-i-svangerskapsomsorgen

2 https://helsedirektoratet.no

3 https://helsedirektoratet.no/publikasjoner/gode-levevaner-for-og-i-svangerskapet
